# Promotion of physical activity interventions for community dwelling older adults: A systematic review of reviews

**DOI:** 10.1371/journal.pone.0180902

**Published:** 2017-07-10

**Authors:** Ania Zubala, Stephen MacGillivray, Helen Frost, Thilo Kroll, Dawn A. Skelton, Anna Gavine, Nicola M. Gray, Madalina Toma, Jacqui Morris

**Affiliations:** 1 Scottish Improvement Science Collaborating Centre, School of Nursing and Health Sciences, University of Dundee, Dundee, Scotland, United Kingdom; 2 Evidence Synthesis Training and Research Group (STAR Group), School of Nursing and Health Sciences, University of Dundee, Dundee, Scotland, United Kingdom; 3 Faculty of Health Sciences and Sport, University of Stirling, Stirling, Scotland, United Kingdom; 4 School of Nursing, Midwifery & Health Systems, University College Dublin, Dublin, Ireland; 5 Institute for Applied Health Research, School of Health and Life Sciences, Glasgow Caledonian University, Glasgow, Scotland, United Kingdom; 6 Nursing, Midwifery and Allied Health Professions Research Unit, Glasgow Caledonian University, Glasgow, Scotland, United Kingdom; Leibniz Institute for Prvention Research and Epidemiology BIPS, GERMANY

## Abstract

**Objectives:**

While there is strong evidence that regular participation in physical activity (PA) brings numerous health benefits to older adults, and interventions to effectively promote PA are being developed and tested, the characteristics and components of the most effective interventions remain unclear. This systematically conducted review of systematic reviews evaluated the effects and characteristics of PA promotion interventions aimed at community dwelling people over 50 years old.

**Methods:**

Major databases were searched for reviews from January 1990 to May 2015. TIDieR guidelines aided data extraction and the ROBIS tool was used to assess the risk of bias. Primary outcomes were objective and self-reported levels of PA. Indicators of psychological wellbeing and participation rates were secondary outcomes.

**Results:**

Of 1284 records identified, 19 reviews met inclusion criteria and eight included meta-analyses. Interventions typically incorporated behaviour change techniques (BCTs) and were delivered as face-to-face, remote, group, individual or as combined interventions. Despite their heterogeneity, interventions often resulted in sustained improvements in PA over the study period, typically at 12 months, and led to improvements in general wellbeing. However, ways to ensure effective maintenance beyond one year are unclear. Certain intervention components were more clearly associated with positive effects (e.g. tailoring promotion strategy with combination of cognitive and behavioural elements, low to moderate intensity activity recommended). We found no evidence that certain other intervention characteristics were superior in achieving positive outcomes (e.g. mode of delivery, setting, professional background of the intervention provider, type of PA recommended).

**Conclusion:**

The evidence suggests that interventions to promote PA among older adults are generally effective but there is uncertainty around the most beneficial intervention components. There are indications that purely cognitive strategies and BCTs might be less suitable for older adults than motivators more meaningful to them, including social and environmental support, and enjoyment coming from being physically active. A whole system-oriented approach is required that is tailored to meet the needs of older adults and aligned with social, individual and environmental factors.

## Introduction

There is considerable and consistent evidence that regular participation in physical activity (PA) has physical and mental health benefits for people of any age [1,2]. In older adults, evidence suggests that exercise reduces risk of cardiovascular disease, osteoporosis, some cancers, falls and cognitive decline [3,4]. Benefits of PA extend to maintaining people’s functional capacity and independence [5]—outcomes particularly relevant to ageing population. There is also substantial evidence that engagement in PA has positive impacts reaching beyond physical health to improved mental wellbeing [6,7,8].

Internationally, guideline recommendations suggest that older people should participate in at least 150 minutes of moderate PA per week [9,10,11], in addition to aerobic and strengthening exercise, and to sit less, to achieve health benefits. However, UK Health surveys from the four UK nations show that 43–77% of men and 48–85% of women aged 65–74 years are not meeting these guidelines and that proportion of inactive older adults increases with increasing age and in those with mobility impairment and disability. In England, the proportion of inactive older people stands at 27% for those aged 65–74 and rapidly increases with age to around 75% for those aged over 85 [12]. In Scotland only 31% of men and 21% of women aged over 75 meet the recommended activity levels [13]. Similar trends are apparent in health surveys worldwide: only 20% of Canadians aged 60–79 meet the guideline of 10,000 steps per day [14] and prevalence of inactivity among Americans aged 65–74 reaches nearly 27% and increases to over 35% for those aged over 75 [15].

Safe, effective, inclusive and sustainable ways to promote PA and to support long-term participation in PA as people age are therefore necessary. These interventions need to be amenable to widespread implementation in a range of health, social care and community settings and be adaptable to meet the needs of older people with mobility restrictions, disabilities, or other limiting health conditions, and those in different socio-demographic groups. To achieve a widespread impact, these interventions should be easily and willingly adopted by older adults living in the community and offer choice to meet both their needs and preferences.

Programmes to increase PA have been developed extensively and reviews of evidence around PA promotion have been undertaken [16,17,18,19,20]. The outcomes reported by these evidence syntheses are generally positive and promising, recognising that interventions to promote PA may be effective and their potential should be further explored. A Cochrane overview of interventions promoting PA engagement for children and adults is currently underway [21]. Despite rich research in the area of PA promotion, published interventions that target older people and those with disabilities are less common. Consequently, with two very recent exceptions [22,23], previous reviews of reviews have not focused specifically on older people and none, to our best knowledge, have systematically examined PA promotion among older adults. Such focus is now vital, given the steadily ageing population [24,25].

Updated evidence on effectiveness of interventions to promote PA in this age group and in community settings is required [26], alongside information on the features of the most successful interventions. This synthesis seeks to identify gaps in current knowledge, contribute to development of novel and effective programmes, and identify which interventions are ready for implementation at scale, with potential to effect sustainable health, wellbeing and fitness benefits across the population of older adults.

### Aims

The current work is a focused structured review of systematic reviews, which aimed to:

1. examine the effectiveness of interventions to promote the uptake and maintenance of PA for community dwelling older adults
2. determine the nature and characteristics of the most effective interventions to promote the uptake and maintenance of PA among community dwelling older adults, including: types of PA promoted, types of the most effective behaviour change techniques (BCTs), outcomes reported, and frameworks and theoretical constructs used to inform these interventions.

## Methods

### Inclusion criteria

Reviews included in this study pertained to PA promotion for older adults and reported PA levels and activities undertaken to influence PA behaviour. Table 1 describes the inclusion and exclusion criteria in more detail.

**Table 1:** Inclusion and exclusion criteria for studies in this review.

Inclusion criteria	Exclusion criteria
1. is a systematic review 2. pertains to implementation of PA promotion 3. includes empirical data related to PA levels 4. concerns community dwelling adults over 50 years of age or at least 60% of included primary studies relate to this age group 5. published in English 6. available electronically	1. not directly related to PA promotion 2. focuses on specific clinical condition or participants in institutionalised care 3. focuses exclusively on effects of exercise and not on interventions to influence PA behaviour

Included reviews could report on interventions delivered by health or exercise professionals or others in health or community settings or fitness facilities. Interventions to promote PA could include any or a combination of: counselling, advice, behaviour change interventions with or without structured PA/exercise. Reviews focused on PA promotion as lifestyle interventions aimed at preventing specific clinical conditions were also included.

Included reviews were required to report on outcomes pertaining to levels of PA, either self-reported (e.g. through the use of an activity diary or self-administered questionnaire) or objectively measured with the use of suitable monitoring devices (e.g. pedometer or accelerometer). Secondary outcomes expected to be reported, but which were not a prerequisite for inclusion, were: a) psychological outcomes (e.g. health-related quality of life, mood, perceived wellbeing), b) participation in PA (e.g. time spent in PA, frequency of participation, drop outs).

### Search methods

**Fig 1:**
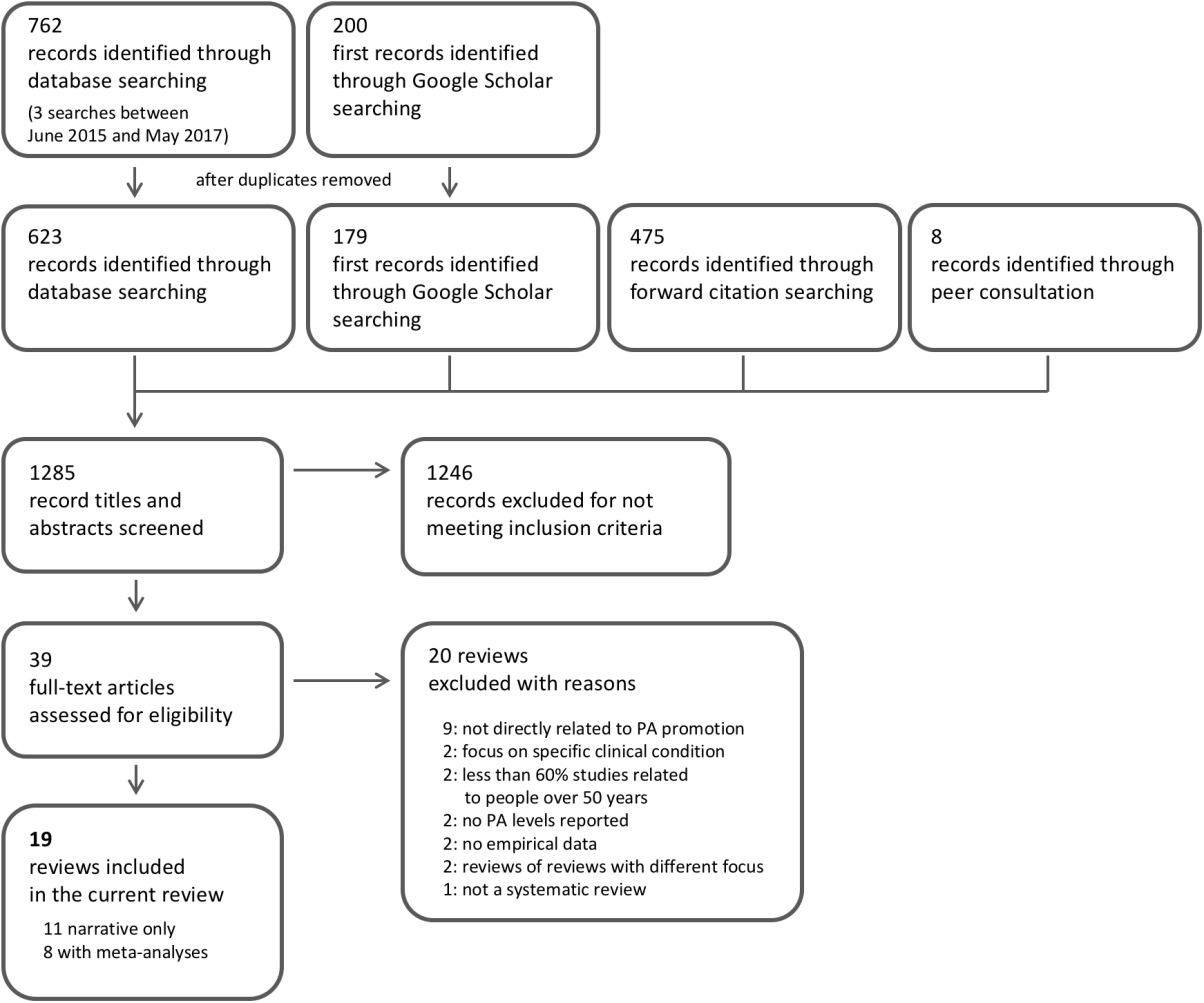
Search flow diagram based on PRISMA guidelines.

The following health-related databases were searched for papers published between January 1990 and May 2017: MEDLINE, CINAHL Plus, PsycINFO, Psychology and Behavioural Sciences Collection, and the Cochrane Library. The search string combined terms relevant to older age and any form of PA and its promotion. In addition, a Google scholar search, forward citation screening, searches of reference lists of included publications, and peer consultation within the research team were used to identify any other relevant articles (see Fig 1 and S3 Table).

### Data extraction and synthesis

Titles and abstracts identified through databases were screened against inclusion criteria independently by two authors (JM and AZ for initial search, SMG and AZ for updated search). Any disagreements were resolved through a third author (HF). Records identified through forward citation and Google Scholar searches were double screened by AG and AZ, with JM acting as arbitrator when needed.

Data were extracted from the included reviews by the first author (AZ) using a data collection form based on the Template for Intervention Description and Replication (TIDieR) [27]. Extracted data included the characteristics of the studies, interventions and outcomes reported in the reviews (see S1 and S2 Tables and Tables 2 and 3). Major conclusions of reviews relevant to the research questions were tabulated and are presented with statistical or narrative evidence on which the conclusions are based. Only the outcomes of interest to this review were recorded.

**Table 2:** Quality assessment of the included reviews based on ROBIS (risk of bias in systematic reviews) tool.

Review & year	ROBIS Assessment				
	Domain 1: Concerns regarding specification of study eligibility criteria	Domain 2: Concerns regarding methods used to identify and/or select studies	Domain 3: Concerns regarding used to collect data and appraise studies	Domain 4: Concerns regarding the synthesis and findings	Risk of bias
Arbesman 2012 [29]	High	High	High	N/A	High
Baxter 2016 [30]	Low	Unclear	Low	N/A	Unclear
Chase 2015* [31]	High	Unclear	High	High	High
Conn 2002* [32]	High	High	High	High	High
Conn 2003 [33]	High	High	High	N/A	High
Foster 2013* [34]	Low	Low	Low	Low	Low
French 2014* [35]	High	High	High	High	High
Geraedts 2013 [36]	Low	Unclear	High	N/A	High
Hobbs 2013* [37]	Unclear	Low	High	High	High
Kassavou 2013* [38]	High	High	High	High	High
King 1998 [39]	High	High	High	N/A	High
Moore 2016 [40]	High	High	High	N/A	High
Müller 2014 [41]	High	High	Low	N/A	High
O'Brien 2015* [42]	Low	Low	Low	Low	Low
Oliveira 2017* [43]	Low	Low	Low	Low	Low
Ostrander 2014 [44]	High	High	High	N/A	High
Stevens 2014 [45]	High	High	Unclear	N/A	High
Van der Bij 2002 [46]	High	High	High	N/A	High
Van der Deijl 2014 [47]	High	High	High	Unclear	High

Highlighted in grey are reviews with low risk of bias.

* reviews with meta-analyses

**Table 3:** Participants’ characteristics and settings.

Review & year	Participants	Location
Arbesman 2012 [29]	*Age*: older adults, mean age over 65	*Setting*: within the scope of practice of occupational therapy
Baxter 2016 [30]	*Age*: age range 40–85 *Gender*: female majority in 45/57 studies (including 13 studies all female) *Ethnic minorities*: large majority white (in 23/24 studies that reported ethnicity) *Other*: participants with no clinical condition or limited mobility	*Setting*: participants’ homes (most common), community (second most common), GP practice *Country*: USA (n = 32), The Netherlands (n = 12), Australia/New Zealand (n = 9)
Chase 2015 [31]	*Age*: older adults, age range 68.5–88, median: 75.35 *Gender*: female majority (median 70%) *Ethnic minorities*: median 15% *Other*: Participants tended to be overweight with a median mean body mass index (BMI) of 27 kg/m2.	*Setting*: home (most common), community (second most common), clinic
Conn 2002 [32]	*Age*: older adults, mean age range 60–77.2 *Gender*: 62% female participants *Ethnic minorities*: 81% participants white (reported in 10/43 studies) *Other*: most studies with participants with no particular health problems (25/43 studies)	*Setting*: home (most common), community centres
Conn 2003 [33]	*Age*: older adults, mean age 65 and older *Gender*: 35% to 100% female participants *Ethic minorities*: 6/17 studies reported *Other*: particular health problems (9/17 studies)	*Setting*: home (most common), also community centres and hospital
Foster 2013 [34]	*Age*: adults, age range 18 to 74 plus *Gender*: 3/11 studies recruited women only *Ethnic minorities*: 7–33% (reported in 7/11 studies) *Other*: free from pre-existing medical conditions	*Setting*: primary health care and the community *Country*: high-income countries (all studies)
French 2014 [35]	*Age*: older adults, mean age 69 years	*Setting*: community centres (n = 9), participants’ homes (n = 5), GP/hospital (n = 3), and others (unclear)
Geraedts 2013 [36]	*Age*: older adults, aged 55 years and older	*Setting*: participants’ homes
Hobbs 2013 [37]	*Age*: older adults, age range 55–70, mean 60.7 *Gender*: 61% female participants	*Setting*: healthcare premises, participants’ homes, university, community *Country*: USA, Belgium, The Netherlands, UK, Finland, New Zealand, Japan, Australia and Canada
Kassavou 2013 [38]	*Age*: older adults, age range 44 to 88, mean 59.8 years	*Setting*: community (n = 17), also hospital (n = 2) *Country*: USA (n = 13), Japan (n = 2), UK, Canada, Australia, China (n = 1 each)
King 1998 [39]	*Age*: older adults, age range 50–91	*Setting*: community centres, retirement complex and home
Moore 2016 [40]	*Age*: older adults *Gender*: 1/7 study recruited only women *Other*: 1/7 study included participants with physical limitation; 2/7 studies included participants recruited from GP clinics	*Setting*: community, rural and regional areas *Country*: USA (n = 3), Australia (n = 2), Canada (n = 1), Taiwan (n = 1)
Müller 2014 [41]	*Age*: older adults, age range 45–89 *Gender*: 60% or more female participants (in 13/16 studies) *Other*: well educated participants, 3/16 studies had 50% participants with higher education	*Setting*: community *Country*: developed countries (all studies)
O'Brien 2015 [42]	*Age*: older adults, age range 55–67.6, mean 60.7 *Gender*: 64% female participants	*Setting*: community *Country*: USA, Europe, New Zealand, Japan, Australia and Canada
Oliveira 2017 [43]	*Age*: older adults, mean age range 60–79 *Gender*: mix gender in 24/27 studies, 3/27 men only *Other*: participants with clinical conditions in 14/27 studies (COPD most common, n = 5)	*Setting*: primary care (n = 8), community (n = 6), hospital (n = 5), outpatient clinic (n = 3), other (n = 5) *Country*: USA (n = 10), The Netherlands (n = 5), Australia (n = 4), UK (n = 2), New Zealand (n = 2), Belgium (n = 2), Italy (n = 1), Canada (n = 1)
Ostrander 2014 [44]	*Age*: older adults, aged over 55 *Ethnic minorities*: primarily white *Other*: populations balanced in terms of gender, income level, education, partnership status, and body mass index	*Setting*: community *Country*: In two studies 100% of participants Dutch
Stevens 2014 [45]	*Age*: older adults, mean age range 65–74 *Gender*: greater number of females in 4/6 studies	*Setting*: general practice (n = 4), local leisure centre (n = 1), by telephone (n = 1) *Country*: New Zealand (n = 2), Australia (n = 1), UK (n = 1), USA (n = 1), Canada (n = 1)—rural and urban settings
Van der Bij 2002 [46]	*Age*: older adults, mean age range 51–88, mean 68 *Gender*: 71% female participants, 1/38 studies recruited women only, 3/38 men only *Ethnic minorities*: large majority white (in 16/17 studies that reported ethnicity) *Other*: mostly healthy, inactive participants; 9/38 studies recruited from care homes and/or GP; most participants well educated, moderate/high incomes	*Setting*: community (majority of studies), also residential or nursing home and primary healthcare facilities *Country*: USA (55% of studies) and Europe (23% of studies)
Van der Deijl 2014 [47]	*Age*: older adults, mean age range 66–84, overall mean 73.8 *Gender*: 70.2% female participants (range 47–89%) in 14/16 studies, 3 studies females only	*Setting*: community

### Assessment of methodological quality

Quality of the included reviews was assessed by two independent reviewers (AG and SMG). Risk of bias was assessed using the ROBIS tool [28], across four domains: (1) study eligibility; 2) identification and selection of studies; 3) data collection and study appraisal; and 4) synthesis and findings, and provides an overall risk of bias within the review as either: high, low or unclear.

## Results

Due to heterogeneity in interventions, comparators and methodology of the included reviews, statistical pooling through meta-analysis was not appropriate. Although the findings of the current review are generally presented in a narrative format, we refer to meta-analyses performed by the included reviews’ authors, whenever available.

### Reviews included and their quality

The database search (performed in June 2015 and subsequently updated to May 5^th^ 2017) yielded 762 records. After deduplication, 623 records were left for screening. A further 200 articles were identified through a search of Google Scholar which after deduplication left 179 for screening. A further 475 articles were identified for screening via forward citation searching and 8 more records via peer consultation (see Fig 1). A total of 1285 records were screened for title and abstracts and 39 unique reviews were retrieved in full and further scrutinised for inclusion. 20 reviews were excluded with reasons which left 19 reviews to be included in this review of reviews [29,30,31,32,33,34,35,36,37,38,39,40,41,42,43,44,45,46,47].

Only three of the 19 included reviews [34,42,43] were judged as having low risk of bias and risk of bias was unclear in one review [30], with the remaining 15 being judged as having high risk of bias (see Table 2). One of the high quality reviews was published by the Cochrane Collaboration [34] and all three reviews included meta-analyses.

For domain 1 (study eligibility), concerns focussed on a lack of clearly defined pre-specified eligibility criteria, restrictions on English language and published journal articles. The main reasons for concern in domain 2 (identification and selection of studies) was a lack of information on methods to reduce error in the study selection process, and limited search strategies. The primary concern in domain 3 (data collection and study appraisal) was lack of formal assessment of risk of bias in the individual studies or use of an inappropriate ROB tool (e.g. calculating a summary score and excluding studies on the basis of this score). For the reviews that did contain a meta-analysis (n = 8) there was considerable heterogeneity in terms of study design and intervention type which was not sufficiently addressed in the synthesis and a lack of assessment of publication bias (e.g. through a funnel plot).

### Characteristics of included reviews

#### Study designs

All reviews (n = 19) were descriptive with eight also including meta-analyses [31,32,34,35,37,38,42,43]. The included reviews reported on 545 studies in 604 papers. However, some studies were included in more than one review, thus the number of unique studies reported was 413 (an overlap of around 24% of studies). Notwithstanding this overlap, all identified reviews were included in the analysis due to their unique focus, methods of analyses and perspectives on the findings from the same studies [48]. Two particular reviews [37,42] originating from one research centre were based on a nearly identical set of identified studies, but both were included due to their different focus (effectiveness of PA promotion and characteristics of successful interventions–both of interest to this review). Additionally, despite a significant overlap in studies within two other reviews [32,33], both were included, as their aims differed and they were together providing complementary information.

The reviews generally included empirical studies, mainly randomised controlled trials (RCTs, 339 studies) and the remainder were quasi-experimental or pre-post studies. Reviews included between 6 and 79 studies, with a mean of 17.8 RCTs per review. One review, though not a review of reviews, also included some evidence coming from systematic reviews and meta-analyses [29] and two reviews did not specify the type of studies included [31,47].

#### Participants

Total numbers of participants were reported in 18 reviews and ranged from 225 to nearly 60,000 per review. Seventeen reviews specifically concerned older people and two focused on adults in general [34,38] although most participants were older adults. The age of participants in the 17 reviews which included only older adults ranged from 40 to 91 years, with mean age ranging from 59.8 to 79 years (reported by 11 reviews).

Of the 14 reviews which specified gender of the participants, nine reported larger numbers of female participants (at least 60% women) [30,31,32,37,41,42,45,46,47], which is representative of a gender split in the older population. Seven reviews mentioned ethnicity [30,31,32,33,34,44,46] and reported that participants were primarily white with a small proportion of ethnic minorities. The majority of reviews either specifically focused on participants with no pre-existing medical condition or did not specify any medical conditions, with the exception of three reviews [32,33,43], which also included patient populations (Table 3).

#### Context/setting

All included reviews focused on older adults living in the community. However, interventions were delivered in diverse settings and included: participants’ homes, general practice and occupational therapy service (Table 3). Most reviews included studies conducted in a range of settings. (In this review home-based interventions refer to instruction or prescription for participants to practice PA at or around home and not to personal supported training delivered by professionals at participants’ homes.)

Eleven reviews reported the country of research, indicating that a vast majority of studies were conducted in developed high-income countries (including USA, UK, Canada, Australia, Japan, China, New Zealand, Taiwan, The Netherlands, Belgium, Italy and Finland).

#### Aim of the reviews

Fourteen reviews aimed to assess the effectiveness of interventions used to increase PA among older adults living in the community. Some of the reviews additionally applied a more specific focus on either the type of intervention (e.g.: remote feedback [36], walking in groups [38], non-face-to-face interventions [41], health coaching [43]); the length of the intervention (e.g. focusing on long-term effects [37,42]); or specific setting (e.g. interventions delivered through a GP service [45], within occupational therapy [29], or in rural or regional areas [40]). Three reviews were concerned with characteristics of interventions or determinants of change [35,40,44]. One review focused specifically on the time around retirement [30] and another on levels of participation in PA programmes [47].

### Description of interventions

Understandably, given the range of purposes mentioned above, there were differences in how interventions were categorised within the reviews. Most reviews reported on diverse, often multimodal interventions and some did not necessarily focus on types of interventions but rather on BCTs used, e.g. [31,32]. Other reviews classified multi-component interventions using group-based, home-based, and education focused categories [46,47], face-to-face and remote qualities of interventions, e.g. [34], outcomes reported, e.g. [37,40] or specific differentiating features (for example, pertaining to individualised feedback and messaging [36,44]).

#### Modes of delivery

Due to heterogeneity and a lack of common approach to categorising interventions in the reviews, they are described here according to the prevailing modes of delivery (see also Table 4). Although interventions could broadly be categorised as face-to-face and non-face-to-face, these categories should be considered a guide only, as they were not always exclusive due to the complexity of the interventions (e.g. some of the remote interventions include an element of direct contact, while educational materials used in many of the face-to-face interventions may be considered remote modes of delivery). The interventions frequently incorporated lifestyle counselling and health education elements and typically took the form of a face-to-face group/individual counselling or training session followed by a scheduled remote contact to encourage further involvement in PA.

**Table 4:** Interventions grouped according to face-to-face or remote modes of delivery.

Mode of delivery	Review	Additional comments on interventions
Face-to-face only	Kassavou 2013* [38]	walking in groups
	Moore 2016 [40]	mostly multi-component interventions
Remote only	Foster 2013* [34]	remote and web 2.0
	Geraedts 2013 [36]	remote feedback
	Müller 2014 [41]	-
	Ostrander 2014 [44]	targeted messaging
Combined (either multi-modal interventions or both face-to-face and remote modes of delivery included)	Arbesman 2012 [29]	activity-based health management
	Baxter 2016 [30]	-
	King 1998 [39]	-
	Hobbs 2013* [37]	mostly multi-modal
	Stevens 2014 [45]	delivered through GP practice
	Van der Bij 2002 [46]	home-based, group-based, education
	Van der Deijl 2014 [47]	home-based, group-based, education
	Chase 2015* [31]	BCTs-focused
	Conn 2002* [32]	BCTs-focused
	Conn 2003 [33]	BCTs-focused
	French 2014* [35]	BCTs-focused
	O’Brien 2015* [42]	BCTs-focused
	Oliveira 2017* [43]	health coaching (face to face and telephone)

* reviews with meta-analyses

*Face-to-face interventions only*: Two reviews included interventions delivered through face-to-face contact only [38,40]; one review [38] focused on interventions to promote walking and another [40] on low- to moderate-intensity exercises and low-impact PA (including walking). Interventions in both reviews were predominantly group-based and consisted of multiple components, including PA itself, as well as education, discussion and counselling elements.

*Remote interventions only*: Four reviews focused entirely on non-face-to-face interventions [34,36,41,44]. Nearly all remote interventions were directed at individuals, instructing them to engage in self-selected PA or a guided exercise programme in their own homes or community. These interventions were primarily based on feedback provided with largely differing frequencies [36,44] and through a variety of modes, including telephone, internet and standard mail. Phone and print materials were the most frequently used modes of delivery in all four reviews, but internet interventions were also emerging [41].

Although remote approaches to PA promotion were the stated subject of the four reviews, three teams of authors [34,36,44] recognised that the primarily non-face-to-face interventions were likely to include an element of direct contact, commonly individual consultation, counselling or introductory session, usually at the beginning of the programme. For example, one review [34] included interventions which had a face-to-face element (the ‘primary dose of intervention’) and the principal remote ‘follow up dose’ (subsequent motivational phone calls or mailing of materials).

Remote interventions often included an educational element–either involving general PA information sent to participants or feedback-based tailored information to provide individualised reports/ leaflets/ exercise plans. A counselling element was also present, e.g. [44].

*Combination of face-to-face and remote interventions*: Thirteen reviews were concerned with either both face-to-face and remote modes of delivery or with complex multimodal interventions [29,30,31,32,33,35,37,39,42,43,45,46,47].

Interventions typically included multiple components and frequently combined education with lifestyle counselling and PA or exercise programme. Two reviews [46,47] categorised interventions as either home-based, group-based or education. The ‘home’ and ‘education’ modalities could be delivered either face-to-face or remotely.

Some authors reported on multimodal delivery format in most included studies [37], others considered PA promotion a part of larger health management and maintenance programmes [29], and some focused on tailored interventions, for which individualised referrals were made through the GP practice [45].

Five reviews focused primarily on BCTs and were not concerned with particular modes of delivery at the study selection stage [31,32,33,35,42] but included diverse multi-modal and multi-component interventions. Other reviews also frequently mentioned the use of BCTs as essential components of the interventions included.

#### Theoretical frameworks

Ten reviews reported on theoretical frameworks purported to underpin the interventions [30,32,33,36,39,40,41,42,43,44]. The most common theoretical framework was Social Cognitive Theory (SCT), mentioned in nine reviews [30,32,33,36,39,41,42,43,44] and reported to have been used in at least 44 studies. The Transtheoretical Model (TTM) was the second most common framework, featuring in six reviews [32,33,41,42,44,43] and at least 21 studies.

#### Intervention components: BCTs

Elements of BCTs were present in all of the discussed interventions and were particularly highlighted in five reviews with a specific focus on BCTs [31,32,33,35,42]. In two reviews the CALO-RE taxonomy [49] was used to code BCTs [35,42]. The five mentioned reviews reported effects of the BCT-based interventions on PA behaviour and one review additionally examined their impact on self-efficacy [35]. Of the five BCT-focused reviews, four performed meta-analyses [31,32,35,42] and their detailed outcomes as well as frequency with which the BCTs were used will be discussed in the outcomes section. One BCT-focused review [33], which did not perform meta-analyses, identified self-monitoring and health education as the most commonly used interventions, followed by goal setting, problem solving, feedback, reinforcement/contingencies, relapse prevention education, and modelling.

Seven other reviews, although not directly concerned with BCTs, reported on some of the techniques used in included interventions. The most commonly reported intervention components were: goal setting [36,37,39,40,41,43,44], self-monitoring [37,39,41,43,44], tailoring [36,41,44], feedback [36,37,39,43], relapse prevention training [39,44], strategies to increase motivation [36,41,43,44] and self-efficacy [36,43,44].

#### Types of physical activity

Eleven reviews did not specify the types of PA promoted [29,30,31,32,34,37,41,42,43,44,45]. In the remaining eight, walking was the most frequently cited form of PA [33,35,36,39,40,46,47] and was the specific focus of one review [38]. In three reviews [33,35,36] at least one third of all included studies were concerned with walking as the main type of PA. Six reviews included other lifestyle PA (e.g. gardening [35], dance [39,46]) and, most commonly, low to moderate intensity activities, for example, a home-based exercise programme [36,39,46,47], tai chi [39,40], aquatic exercises [40] and yoga [46]. Although less vigorous activities were generally most common, reviews also mentioned aerobic-type exercise including jogging [35,36,39,46].

Some reviews made a distinction between interventions with a ‘prescribed’ or structured format and interventions in which participants were able to self-select their preferred PA type. In only one review [38] the type of PA was clearly defined for all studies and five reviews included both prescribed and self-select activities [32,34,36,40,45].

#### Intervention providers

Just as interventions varied significantly, their providers included a range of professionals within the fields of: healthcare (GPs, nurses, physicians, occupational therapists, physiotherapists, health visitors), PA (exercise counsellors, PA coaches, health and fitness professionals, community exercise instructors, certified exercise trainers) and education (trained health educator, physical educator). Some interventions were reported to have been facilitated by a trained member of a research team [35,37,43] or a graduate student [33,46] and there was also mention of an intervention offered through an ‘automated computer’ [34]. As expected in interventions offered largely in the community and focusing on PA promotion rather than exercise classes, most reviews reported on participants taking an active role in the actual ‘delivery’ of the intervention. Elements of self-help and/or self-administered exercise under instruction [37,42] were present in many if not the majority of the studies. In some cases, PA was encouraged or sessions facilitated by peer mentors or lay leaders [30,33,35,38].

#### Intervention tailoring

Six reviews provided information on intervention tailoring [29,33,37,41,44,45], with the majority of interventions individualised to some degree. One review [37], for example, stated that some interventions provided participants with tailored exercise prescriptions, while others (educational interventions) offered information specific to the individual, taking into account their health, environment, opportunities and goals. The remaining thirteen reviews did not provide details sufficient to assess the presence or degree of tailoring.

#### Dose and duration

Reported interventions lasted between four weeks and three years, with durations of between three months and 12 months being most common. Shorter interventions of between four and 12 weeks were slightly less common, and duration of over 12 months was significantly less common. The longest reported intervention lasted for 90 months and was an ongoing PA programme [46], while the shortest intervention lasted for one day only and involved four different text messages sent to participants over the course of a single day [44].

The frequency of sessions varied largely between different types of interventions and within similar intervention type or component. In interventions which included face-to-face educational and lifestyle counselling component (e.g. lectures, motivational sessions), sessions were commonly offered on a weekly basis [29,33]. In interventions which actually monitored or at least recommended activity, participants were asked to engage in PA between one and seven times per week, with two to five times per week being the most common frequency of activity prescription [29,33,36,38,45,46]. Some reviews reported on interventions which incorporated daily activity [36,38], while the least frequent intervention reported activity once a month for a relatively longer period of time (90 to 120 minutes) than in the case of more frequent sessions [38]. In most interventions activity sessions lasted for between 10 and 90 minutes, with a range of 20 to 60 minutes being most common. Some interventions began with shorter sessions (e.g. 10–15 minutes) and eventually progressed to being active for longer periods of time (e.g. 45 or 60 minutes).

In case of remote feedback-based interventions (i.e. phone calls and text messages) there was a substantial variability across studies, with participants receiving messages and/or calls at either regular or varied intervals [44]. The frequency of contacts could be anything between three times per day (e.g. [44]) to once every two months (e.g. [45]). However, there seemed to be a common pattern of contacts being more frequent at the beginning of the intervention (often weekly for the first month) and maintained through less frequent calls/messages (biweekly/monthly) over the course of the intervention (e.g. [34]). Overall number of messages or phone calls was rarely reported in the reviews and ranged between three and 18 contacts [30,41].

Three reviews concerned with primarily with multimodal interventions [31,37,42] reported that number of sessions or contacts within an intervention ranged from one to 228 sessions and averaged between 15 and 37 sessions per intervention.

### Outcomes reported in reviews

**Fig 2:**
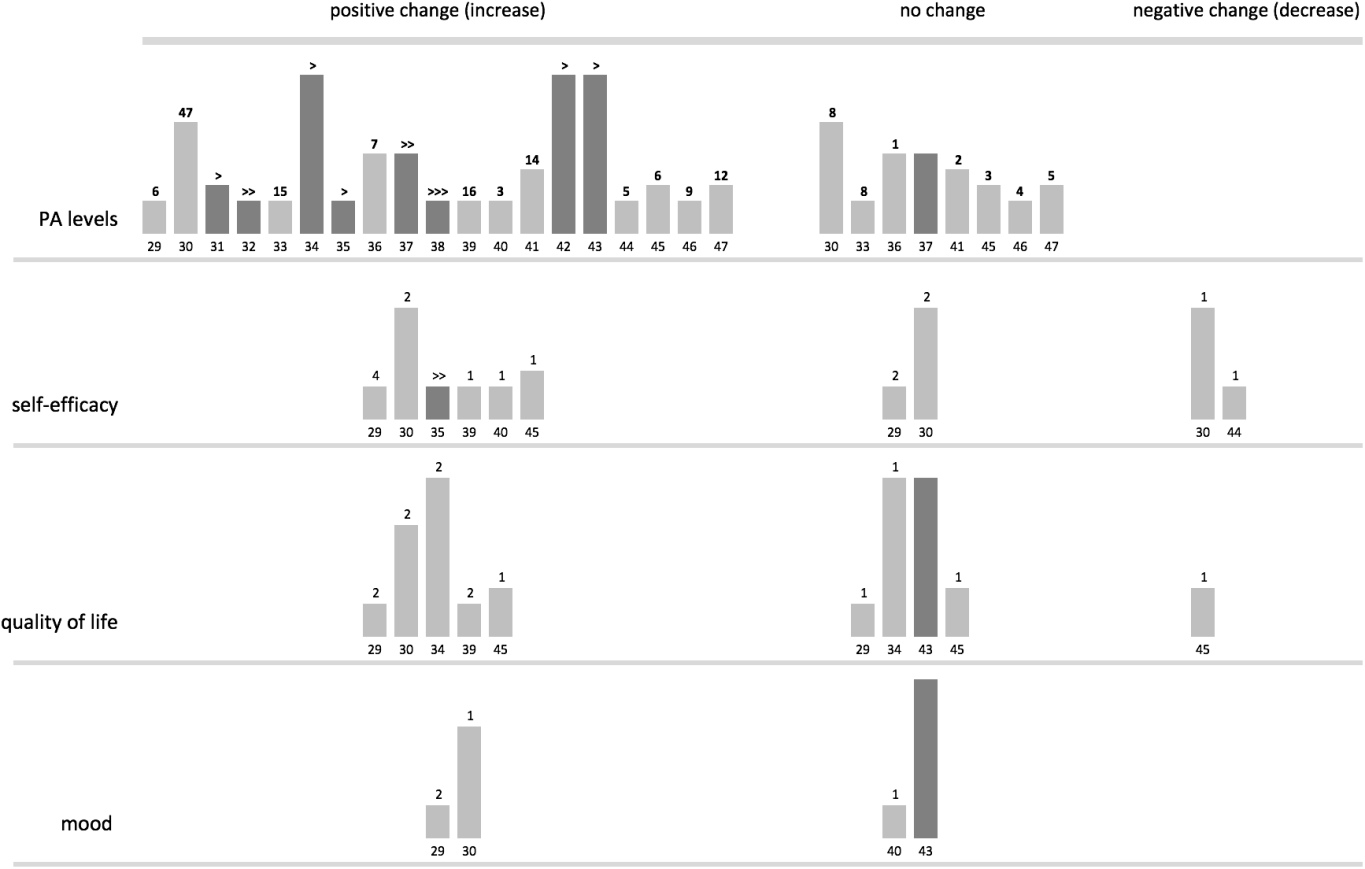
Harvest plot: Evidence for PA and psychological outcomes. Columns represent individual reviews with reference numbers below. Column height represents risk of bias assessed on four domains—higher columns represent lower risk of bias. Lighter shade designates narrative evidence, darker shade designates evidence from meta-analysis. Numbers above columns indicate number of studies reporting effect (for narrative reviews). Arrows indicate effect size (for meta-analyses: > small effect, >> mixed effects, >>> moderate effect).

**Fig 3:**
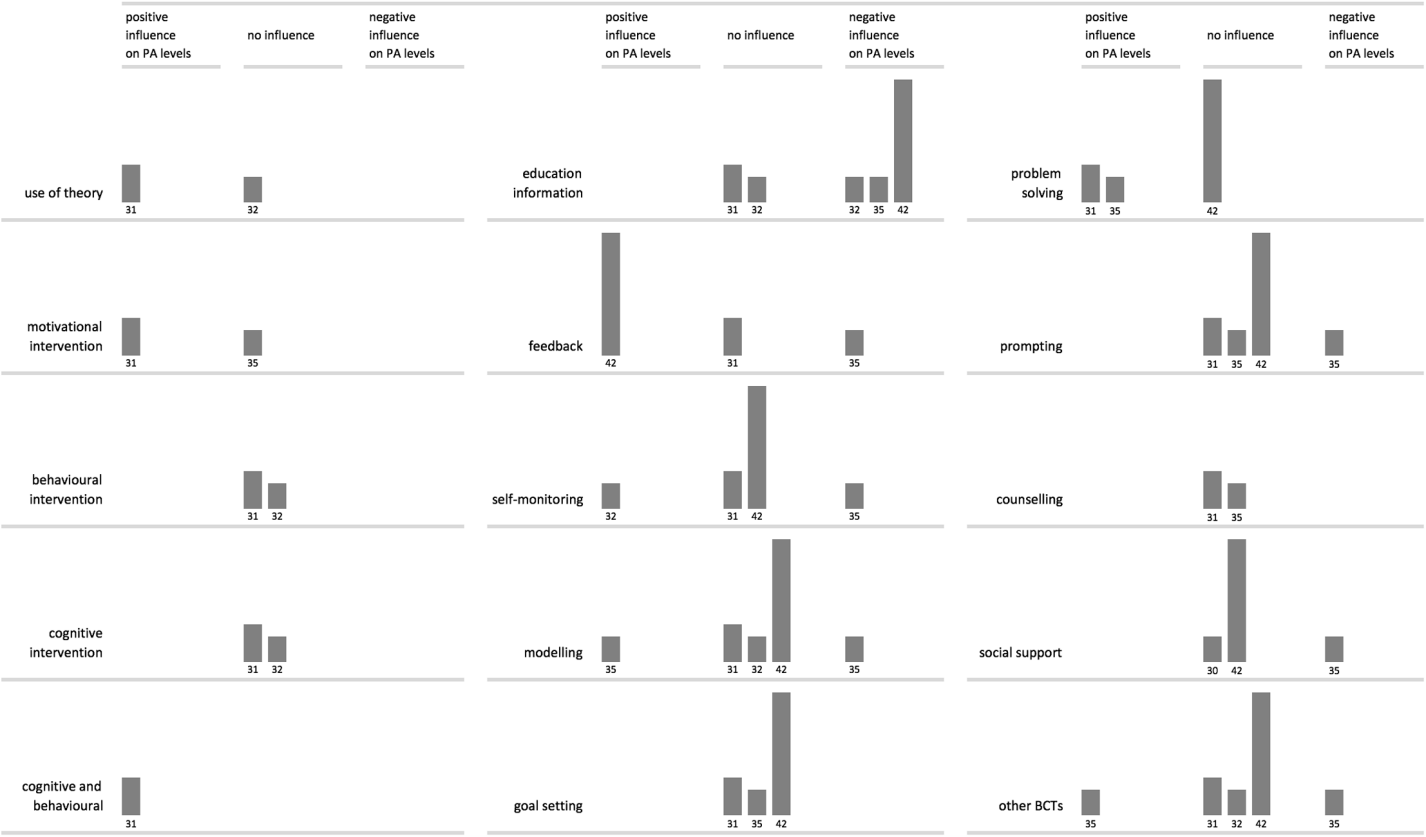
Harvest plot: Behaviour change techniques and their influence on PA levels. Columns represent individual reviews with reference numbers below. Column height represents risk of bias assessed on four domains—higher columns represent lower risk of bias.

All reviews included outcomes pertaining to PA levels and some reviews reported on psychological, physiological and functional outcomes, as well as on participation levels. For the purpose of the current review we have looked at: 1) PA outcomes, 2) psychological outcomes, 3) participation and adherence rates. Results from the eight reviews including meta-analyses [31,32,34,35,37,38,42,43] are described first, followed by findings from the eleven narrative reviews [29,30,33,36,39,40,41,44,45,46,47]. Meta-analyses are presented in two sections focusing on: a) effectiveness rates, and b) moderator analyses. The included reviews typically compared treatment groups to control groups, in which participants did not receive any intervention, received treatment as usual (in case of studies in health care settings) or were offered an alternative intervention (e.g. education alone). In addition to tables we provide harvest plots [50,51] as graphical representation of the evidence (Figs 2 and 3).

Eight reviews performed formal meta-analyses of the results of the studies they included [31,32,34,35,37,38,42,43]. One of these reviews focused on face-to-face walking interventions only [38], one was specifically interested in remote interventions [34] and the rest included studies with both modes of delivery. The eight reviews explored comparisons of interventions to control conditions, including either usual care, no intervention or intervention different to PA promotion. When data from two groups were not available, some reviews performed single group comparisons (e.g.[31]).

Eleven narrative reviews reported results pertaining to PA levels, psychological outcomes, participation and characteristics of effective interventions. One review which performed meta-analysis on PA levels [34] is also included in this section, as it used a descriptive only way of reporting on quality of life measurement.

#### Reviews including meta-analyses: PA levels

All of the reviews’ authors concluded that interventions aiming to increase PA were effective under differing conditions and with differing effect sizes observed (see Table 5).

**Table 5:** Physical activity levels in reviews with meta-analyses, including sub-group analyses for short/long term outcomes.

Effectiveness of interventions: PA levels (reviews with meta-analyses)										
review	time at measurement		studies (participants)	outcome measured	effect size (d / OR)	95% CI	significance (p value)		effect	ROB
Chase 2015 [31]	not reported		46 (10,186)	PA levels (two-group, treatment vs control comparison, post-intervention)	d = 0.18	0.10–0.26	p < .001		small	high
	not reported		33 (3653)	PA levels (single group, pre-post intervention)	d = 0.23	0.15–0.31	p < .001		small	
Conn 2002 [32]	not reported		43 (33,090)	PA levels	d = 0.26	± .05	not reported		small	high
	sub-group	up to 90 days	16	PA levels, short-term outcome (up to 90 days)	d = 0.42	± .16	not reported	p < .05 (sub-group difference)	moderate	
		over 180 days	13	PA levels, long-term outcome (over 180 days)	d = 0.22	± .12	not reported		small	
Foster 2013* [34]	24 months		1 (1049)	PA levels self-reported (continuous outcome measure)	d = 0.20	0.08–0.32	not reported		small	low
	12 months		9 (4547)	PA levels self-reported (continuous outcome measure)	d = 0.20	0.11–0.28	not reported		small	
	12 months		1 (1089)	PA levels self-reported (dichotomous outcome measure)	OR = 1.73	1.34–2.21	not reported		small	
	24 months		1 (1088)	PA levels self-reported (dichotomous outcome measure)	OR = 1.33	1.03–1.70	not reported		small	
Hobbs 2013 [37]	12 months		11	PA duration self-reported (continuous outcome measure)	d = 0.19	0.10–0.28	p < .0001		small	high
	12 months		3	PA duration self-reported (dichotomous outcome measure)	OR = 1.63	1.06–2.49	p = .02		small	
	18 months		not reported	PA duration self-reported (continuous outcome measure)	d = 0.10	-0.08–0.29	n/a		no effect	
	18 months		1	PA duration self-reported (dichotomous outcome measure)	OR = 1.21	0.95–1.54	not reported		no effect	
	24 months		4	PA duration self-reported (continuous outcome measure)	d = 0.07	-0.06–0.20	n/a		no effect	
	24 months		1	PA duration self-reported (dichotomous outcome measure)	OR = 1.33	1.03–1.70	not reported		small	
	12 months		4	PA levels objective: step-count	d = 1.08	0.16–1.99	p = .02		large	
	18 months		1	PA levels objective: step-count	d = 0.38	0.16–0.60	not reported		moderate	
	24 months		1	PA levels objective: step-count	d = -0.01	-0.41–0.40	n/a		no effect	
	12 months		1	PA levels objective: accelerometer	d = 0.18	-0.18–0.55	not reported		small	
	24 months		1	PA levels objective: accelerometer	d = -0.01	-0.42–0.40	n/a		no effect	
Kassavou 2013 [38]	varied		19 (4752)	PA levels self-reported and objective	d = 0.52	0.32–0.71	p < .0001		moderate	high
	sub-group	up to 6 months	13 (2992)	PA levels, short-term outcome (up to 6 months)	d = 0.45	0.25–0.65	p < .0001	p = 0.0004 (sub-group difference)	moderate	
		over 6 months	6 (1580)	PA levels, long-term outcome (over 6 months)	d = 0.66	0.22–1.10	p < .001		moderate	
O'Brien 2015* [42]	12 months (14 s.) to 36 months		19 (10,423)	PA levels self-reported	d = 0.29	0.19–0.40	p < .01		small	low
Oliveira 2017* [43]	immediately post-intervention		27 (5,803)	PA levels (self-reported and objective)	d = 0.27	0.18–0.37	p < .001		small	low
French 2014 [35]	not reported		16	PA levels	d = 0.14	0.09–0.20	p < .001		small	high

Highlighted in orange are significant effects (colour intensity indicates effect size)

* reviews with low risk of bias (ROB)

The review which focused on walking interventions [38] reported a medium-sized positive effect (d = 0.52) on PA and the largest pooled effect reported by any of the included reviews. This effect was also moderate for short term outcomes up to six months (d = 0.45) and even larger for outcomes measured beyond six months (d = 0.66). The difference between the two sub-groups was significant (p = 0.0004). In another review comparing short and longer term outcomes [32], multi-modal interventions were effective in both groups, but the effect size was moderate for outcomes up to 90 days (d = 0.42) and only small for outcomes over 180 days (d = 0.22), with a group difference significant at p < 0.05 level.

One review [37] reported positive small to moderate effects of multi-modal interventions on outcomes measured at 12 months (d = 0.19, p < 0.0001), but no significant effects at 24 months, while another review, focused on remote interventions [34], calculated comparably small effects for outcomes at 12 and 24 months (d = 0.20, for both conditions). In reviews which either did not specify times at measurement, took post-intervention measurement only or calculated combined effects [31,32,35,38,42,43], effects were positive and small to moderate (SMD varying between 0.14 to 0.52), suggesting that the multi-modal interventions examined were found to be effective at promoting PA behaviour among older adults.

#### Reviews including meta-analyses: Psychological outcomes

One review [35] calculated the pooled effect of 24 studies with multi-modal interventions including BCTs on self-efficacy. A moderate positive effect (d = 0.37) was found (see Table 6). Another review [43] found no evidence of a differential effect of health coaching in improving quality of life (8 studies, d = 0.07) or mood (5 studies, d = 0.02) based on pooled calculations of measurements taken immediately after the intervention.

**Table 6:** Psychological outcomes (self-efficacy levels) in a review with meta-analyses.

Effectiveness of interventions: psychological outcomes (reviews with meta-analyses)								
review	time at measurement	studies	outcome measured	effect size (d value)	95% CI	Significance (p value)	effect	ROB
French 2014 [35]	not reported	24	self-efficacy levels	d = 0.37	0.22–0.52	p < .001	moderate	high
Oliveira 2017* [43]	immediately post-intervention	8	quality of life	d = 0.07	-0.06–0.20	p < 0.05	no effect	low
	immediately post-intervention	5	mood	d = 0.02	-0.12–0.16	p = 0.83	no effect	

Highlighted in orange is significant effect

* review with low risk of bias (ROB)

#### Reviews including moderator analyses: Intervention characteristics

Six reviews [31,32,34,38,42,43] performed moderator analyses related to the features of interventions examined and their impact on PA levels. These are categorised in the current review according to the characteristics of the participants, intervention providers, the setting and mode of intervention (see Table 7).

**Table 7:** Moderator analyses: Intervention characteristics—participants, interventionist, setting, mode, other characteristics.

Moderator analyses: Intervention characteristics (participants, interventionist, setting, mode, other)								
category	sub-category	review	moderator analyses	present		absent		significance
				SMD (95% CI)	studies (participants)	SMD (95% CI)	studies (participants)	
participants	health condition	Chase 2015 [31]	history of chronic illness	d = 0.11	35	d = 0.30	16	p = 0.03
		Oliveira 2017* [43]	health condition	d = 0.32 (0.19–0.45)	14	d = 0.23 (0.10–0.36)	13	ns
		Conn 2002 [32]	patient sample	d = 0.39 (± .12)	18	d = 0.26 (± .10)	25	p < 0.05
		Kassavou 2013 [38]	women participants only	d = 0.18 (0.03–0.33)	6 (702)	d = 0.61 (0.35–0.88)	13 (3870)	p < .0001
		Kassavou 2013 [38]	participants up to 59 years (present) vs over 60 years (absent)	d = 0.48 (0.27–0.69)	12 (2548)	d = 0.57 (0.17–0.98)	7 (2024)	p = 0.05
intervention provider / facilitator		Conn 2002 [32]	intense contact with interventionists	d = 0.44 (± .13)	14	d = 0.19 (± .12)	14	p < 0.01
		Chase 2015 [31]	exercise specialist interventionist	d = 0.14	20	d = 0.20	33	ns
		Foster 2013* [34]	interventionist: health professional	d = 0.21 (0.09–0.34)	2 (1067)	d = 0.19 (0.09–0.30)	7 (3480)	ns
		Kassavou 2013 [38]	interventionist: professional	d = 0.51 (0.23–0.79)	11 (1729)	d = 0.52 (0.25–0.79)	8 (2843)	ns
setting		Conn 2002 [32]	centre-based (present) vs home based (absent)	d = 0.47 (± .16)	15	d = 0.24 (± .08)	28	p < 0.001
		Chase 2015 [31]	delivered in more than one setting	d = 0.29	16	d = 0.13	37	ns
	home	Chase 2015 [31]	delivered at participant's house	d = 0.20	25	d = 0.16	28	ns
		O'Brien 2015* [42]	home setting	d = 0.28 (0.17–0.39)	14	d = 0.37 (0.03–0.70)	5	ns
		Chase 2015 [31]	delivered in a community setting	d = 0.17	15	d = 0.18	38	ns
	health care	Chase 2015 [31]	delivered in a clinic setting	d = 0.09	8	d = 0.20	45	ns
		O'Brien 2015* [42]	health care setting	d = 0.36	10	d = 0.23	9	ns
mode of delivery	mail	Chase 2015 [31]	use of mailed materials	d = 0.34	10	d = 0.14	43	p = 0.03
		O'Brien 2015* [42]	print material delivery	d = 0.14 (0.07–0.22)	8	d = 0.48 (0.28–0.67)	11	p < 0.01
		Chase 2015 [31]	use of audio-visual media	d = 0.48	5	d = 0.14	48	p = 0.01
	group	Chase 2015 [31]	group setting	d = 0.19	34	d = 0.16	19	ns
		Conn 2002 [32]	group setting	d = 0.37 (± .12)	23	d = 0.22 (± .09)	18	p < 0.05
		Chase 2015 [31]	use of written materials	d = 0.23	30	d = 0.08	23	ns
		Chase 2015 [31]	use of discussion	d = 0.21	32	d = 0.11	21	ns
		Chase 2015 [31]	use of lecture	d = 0.11	21	d = 0.22	32	ns
	phone	Chase 2015 [31]	phone mediated	d = 0.21	19	d = 0.15	34	ns
		O'Brien 2015* [42]	telephone delivery	d = 0.29 (0.16–0.42)	10	d = 0.32 (0.12–0.51)	9	ns
		Oliveira 2017* [43]	telephone (present) vs face to face (absent)	d = 0.21 (0.11–0.32)	18	d = 0.41 (0.25–0.58)	9	p < 0.05
		Chase 2015 [31]	face to face interaction	d = 0.18	44	d = 0.16	9	ns
		Conn 2002 [32]	mediated delivery (phone, mail)	d = 0.21 (± .10)	12	d = 0.27 (± .11)	31	ns
other intervention characteristics	intensity	Conn 2002 [32]	recommended moderate-intensity PA (present) vs low-intensity PA (absent)	d = 0.58 (± .17)	10	d = 0.26 (± .14)	13	p < 0.01
		Conn 2002 [32]	intensity level recommended	d = 0.39 (± .12)	23	d = 0.25 (± .10)	20	p < 0.05
		Conn 2002 [32]	target PA behaviour only (present) vs target multiple health behaviours (absent)	d = 0.38 (± .11)	18	d = 0.23 (± .12)	15	p < 0.01
	PA type	Foster 2013* [34]	PA type specified	d = 0.36 (0.05–0.66)	1 (189)	d = 0.19 (0.10–0.27)	8 (4358)	ns
		Conn 2002 [32]	walking recommended	d = 0.40 (± .18)	11	d = 0.29 (± .10)	32	ns
		Foster 2013* [34]	prescribed PA: human generated (present) vs computer generated (absent)	d = 0.22 (0.10–0.34)	5 (2491)	d = 0.18 (0.04–0.33)	4 (2056)	ns
		Foster 2013* [34]	use of pedometer	d = 0.16 (0.05–0.27)	3 (1456)	d = 0.23 (0.11–0.35)	6 (3091)	ns

Highlighted in green are significant effects

* reviews with low risk of bias (ROB)

Participants: In one review [31] interventions tested among healthy subjects were found to be more effective than those aimed at chronically ill participants. In contrast, another review [32] found significantly larger effect sizes for patient populations (cardiac problems, arthritis and diabetes) than for healthy participants and another review [43] found no differentiating effect of health conditions. In another paper [38] larger effect sizes were found for interventions directed at mixed gender groups than at women participants only and at adults aged over 60 years than at younger adults.

Intervention provider: In one review, interventions which featured higher levels of programme intensity (intense contacts with intervention providers) had higher effect sizes than those that featured relatively less intensive contact [32]. Three reviews calculated that interventions delivered by non-professionals had similar effect sizes to interventions facilitated by professionals, including health practitioners and exercise specialists [31,34,38].

Setting: One review found that studies testing centre-based activity had larger positive effect sizes than studies promoting home-based activity [32]. In another two reviews neither types of settings [31,42] nor their number [31] appeared to affect PA levels.

Mode of delivery: In two reviews the use of printed and mailed materials led to larger effect sizes compared to not using this delivery mechanism, but the effect was positive in one review [31] and negative in another [42]. Interventions that employed audio-visual media had a more positive effect on PA levels than those that did not use this form of delivery [31]. Using other modes, including written materials, discussion, lectures and phones led to similar effect sizes as not using these methods in three reviews [31,32,42]. Similarly, no difference was found in interventions which were and were not delivered face-to-face [31]. However, review that compared telephone and face to face delivery [43] found that, while both were effective, face to face interventions led to stronger positive effects. Of the two reviews that examined the effect of interventions offered to groups and individual people, one indicated that interventions directed at individuals resulted in smaller effect sizes than interventions delivered to groups [31], the other found no differences in effect [32].

Other intervention characteristics: One review which examined the effects of PA intensity [32] concluded that studies recommending moderate-intensity PA recorded significantly larger effect sizes than those recommending low-intensity PA. Additionally, interventions that made any recommendation on intensity levels had larger positive effects than interventions without an intensity recommendation. In the same review studies focusing on PA promotion specifically had higher effect sizes than studies designed to change multiple health behaviours [32]. Two reviews examined the impact of recommended PA activity type [32,34]: recommended walking was not found to lead to statistically different effect sizes compared to no specific recommendation [32] and there was no statistical difference between interventions that specified PA and those that allowed participants a choice [34]. Similarly, no difference in effects was reported for studies that generated their PA prescriptions by computers and by humans and for studies that used and did not use pedometers as part of intervention [34].

In addition to the described moderator analyses of dichotomous variables, one review [31] performed analyses of continuous variables, including: duration of intervention, length and number of sessions, participant baseline BMI and age and percentage of women. The calculations concluded that the interventions were similarly effective regardless of the different variables examined.

#### Reviews including moderator analyses: BCTs

Four reviews [31,32,35,42] examined the impact of BCTs on PA levels. Two of these [35,42] used CALO-RE taxonomy [49], enabling direct comparisons. Other reviews did not specify the background for the adopted nomenclatures. Therefore, the current review attempted to categorise the BCTs included in moderator analyses of all four reviews for a clearer and more systematic report (see Table 8).

**Table 8:** Moderator analyses: Behaviour change techniques–BCTs.

Moderator analyses: Behaviour Change Techniques (BCTs)								
category	BCT *(CALO-RE taxonomy in italics)*	review	present SMD (95% CI)	studies (participants)	absent SMD (95% CI)	studies (participants)		significance
Theory and intervention type	Use of theory	Chase 2015 [31]	d = 0.28	28	d = 0.05	25		p < 0.01
		Conn 2002 [32]	d = 0.25 (± .13)	15	d = 0.28 (± .08)	28		ns
	Motivational-type intervention	Chase 2015 [31]	d = 0.20	46	d = -0.15	7		p = 0.02
	*Motivational interviewing*	French 2014 [35]	d = 0.22	2 (1103)	d = 0.17	14 (4478)		ns
	Behavioural-type intervention components only	Chase 2015 [31]	d = 0.09	12	d = 0.20	41		ns
	Behaviour modification	Conn 2002 [32]	d = 0.34 (± .14)	17	d = 0.31 (± .10)	26		ns
	Cognitive-type intervention components only	Chase 2015 [31]	d = 0.03	5	d = 0.18	48		ns
	Cognitive modification	Conn 2002 [32]	d = 0.25 (± .13)	12	d = 0.34 (± .10)	31		ns
	Combination cognitive- and behavioural-type intervention	Chase 2015 [31]	d = 0.23	36	d = 0.02	17		p = 0.03
Feedback	Feedback	Chase 2015 [31]	d = 0.23	19	d = 0.14	34		ns
	*Provide feedback on performance*	French 2014 [35]	d = 0.15	6 (4095)	d = 0.27	10 (1486)		p < 0.05
		O'Brien 2015* [42]	d = 0.40 (0.24–0.56)	11	d = 0.19 (0.05–0.32)	8		p < 0.05
Self-monitoring	Self-monitoring	Chase 2015 [31]	d = 0.24	30	d = 0.10	23		ns
		Conn 2002 [32]	d = 0.39 (± .14)	14	d = 0.30 (± .10)	27		p < 0.01
	*Prompt self-monitoring of behavioural outcome*	O'Brien 2015* [42]	d = 0.43 (-0.04–0.89)	5	d = 0.26 (0.17–0.35)	14		ns
	*Prompt self-monitoring of behaviour*	O'Brien 2015* [42]	d = 0.28 (0.15–0.42)	13	d = 0.33 (0.13–0.52)	6		ns
		French 2014 [35]	d = 0.13	9 (3703)	d = 0.24	7 (1878)		p < 0.05
Modelling	Role modelling	Chase 2015 [31]	d = 0.25	6	d = 0.17	47		ns
	*Provide normative information about others’ behaviour*	French 2014 [35]	d = 0.06	4 (3590)	d = 0.30	12 (1991)		p < 0.001
	Social modelling	Conn 2002 [32]	d = 0.28 (± .20)	12	d = 0.33 (± .09)	29		ns
	*Model/demonstrate the behaviour*	French 2014 [35]	d = 0.35	7 (1413)	d = 0.08	9 (4168)		p < 0.001
		O'Brien 2015* [42]	d = 0.55 (0.18–0.91)	5	d = 0.22 (0.12–0.32)	14		ns
Social support	*Plan social support / social change*	French 2014 [35]	d = 0.07	10 (4317)	d = 0.40	6 (1264)		p < 0.001
		O'Brien 2015* [42]	d = 0.20 (0.11–0.29)	9	d = 0.38 (0.18–0.58)	10		ns
	Social support	Conn 2002 [32]	d = 0.29 (± .21)	11	d = 0.31 (± .09)	30		ns
Goal setting	Goal setting by the interventionist	Chase 2015 [31]	d = 0.25	8	d = 0.16	45		ns
	Goal setting by the participant	Chase 2015 [31]	d = 0.27	18	d = 0.12	35		ns
	*Goal setting (outcome)*	O'Brien 2015* [42]	d = 0.28 (0.13–0.42)	5	d = 0.30 (0.16–0.44)	14		ns
	*Goal setting (behaviour)*	O'Brien 2015* [42]	d = 0.27 (0.11–0.55)	13	d = 0.33 (0.15–0.39)	6		ns
		French 2014 [35]	error in table, but no significant effect reported in text					
	*Prompt review of behavioural goals*	O'Brien 2015* [42]	d = 0.33 (0.16–0.50)	5	d = 0.28 (0.15–0.42)		14	ns
		French 2014 [35]	d = 0.24	6 (991)	d = 0.14		10 (4590)	ns
Problem solving	Barriers management	Chase 2015 [31]	d = 0.30	20	d = 0.08		33	p < 0.05
	*Barrier identification / problem solving*	O'Brien 2015* [42]	d = 0.20 (0.11–0.29)	10	d = 0.38 (0.17–0.60)		8	ns
		French 2014 [35]	d = 0.27	10 (1257)	d = 0.15		6 (4324)	p < 0.05
	Problem solving	Chase 2015 [31]	d = 0.30	22	d = 0.08		31	p < 0.05
Education	Patient education	Chase 2015 [31]	d = 0.29	10	d = 0.15		43	ns
	Health education	Chase 2015 [31]	d = 0.20	15	d = 0.17		38	ns
		Conn 2002 [32]	d = 0.26 (± .09)	30	d = 0.59 (± .17)		11	p < 0.001
	*Provide information on consequences of behaviour to the individual*	O'Brien 2015* [42]	d = 0.15 (0.09–0.21)	10	d = 0.57 (0.32–0.82)		8	p = 0.001
		French 2014 [35]	d = 0.10	6 (3196)	d = 0.20		10 (2385)	p < 0.05
	*Provide information on consequences of behaviour in general*	French 2014 [35]	d = 0.16	11 (2725)	d = 0.20		5 (2856)	ns
	*Provide information on where and when to perform the behaviour*	French 2014 [35]	d = 0.04	3 (2299)	d = 0.21		13 (3282)	p < 0.001
		O'Brien 2015* [42]	d = 0.15 (0.08–0.22)	6	d = 0.38 (0.21–0.54)		13	p < 0.05
	*Provide instruction on how to perform the behaviour*	O'Brien 2015* [42]	d = 0.42 (0.19–0.65)	7	d = 0.23 (0.12–0.35)		12	ns
		French 2014 [35]	d = 0.15	11 (3888)	d = 0.18		5 (1693)	ns
Prompting	Prompting	Chase 2015 [31]	d = 0.16	10	d = 0.19		43	ns
	*Prompt practice*	O'Brien 2015* [42]	d = 0.55 (0.16–0.95)	5	d = 0.23 (0.13–0.32)		14	ns
		French 2014 [35]	d = 0.14	13 (5387)	d = 0.38		3 (194)	p < 0.05
	*Use of follow-up prompts*	O'Brien 2015* [42]	d = 0.25 (0.13–0.37)	11	d = 0.38 (0.18–0.59)		8	ns
		French 2014 [35]	d = 0.18	2 (449)	d = 0.14		14 (5132)	ns
	*Prompt use of imagery*	French 2014 [35]	d = 0.20	2 (91)	d = 0.18		14 (5490)	ns
Counselling	*Stress management/emotional control training*	French 2014 [35]	d = 0.09	3 (547)	d = 0.15		13 (5034)	ns
	Counselling	Chase 2015 [31]	d = 0.20	25	d = 0.14		28	ns
other BCTs	*Set graded tasks*	O'Brien 2015* [42]	d = 0.40 (0.22–0.59)	11	d = 0.20 (0.09–0.32)		8	ns
		French 2014 [35]	d = 0.44	2 (82)	d = 0.17		14 (5499)	ns
	Supervised exercise intervention	Chase 2015 [31]	d = 0.16	27	d = 0.19		26	ns
	Behavioural target	Chase 2015 [31]	d = 0.22	20	d = 0.16		33	ns
	Self-efficacy enhancement	Chase 2015 [31]	d = 0.24	17	d = 0.13		36	ns
	Referral to community resources	Chase 2015 [31]	d = 0.12	7	d = 0.19		46	ns
	Exercise prescription	Conn 2002 [32]	d = 0.40 (± .16)	17	d = 0.27 (± .09)		24	ns
	*Action planning*	French 2014 [35]	d = 0.10	7 (4412)	d = 0.30		9 (1169)	p < 0.01
	*Provide rewards contingent on effort or progress towards behaviour*	French 2014 [35]	d = 0.08	2 (415)	d = 0.15		14 (5166)	ns
	*Provide rewards contingent on successful behaviour*	French 2014 [35]	d = 0.27	3 (696)	d = 0.13		13 (4885)	p < 0.05
	*Relapse prevention/coping planning*	French 2014 [35]	d = 0.09	3 (2644)	d = 0.19		13 (2937)	p < 0.05
	*Prompting focus on past success*	French 2014 [35]	d = 0.11	3 (394)	d = 0.16		13 (5187)	ns

Highlighted in purple are significant effects

* review with low risk of bias (ROB)

Forty-four of the total 66 comparisons in the reviews resulted in non-significant effects. We identified 51 different BCTs. Fifteen of them were mentioned in more than one review. Effects of the 15 BCTs for which more than one comparison was available were predominantly inconsistent, with one case of a contradicting result (for BCT ‘feedback’) and only one case of significant effect confirmed by two reviews (for BCT ‘Provide information on consequences of behaviour to the individual’).

Theory and intervention type: Of the two reviews which looked at the use of theory [31,32], one reported that theory-based interventions had larger effect sizes than interventions without a stated theoretical basis [31], the other found no link between the use of a theoretical framework and effectiveness [32]. While in one review motivational-type interventions were reported to have been more effective than interventions without motivational element [31], another review found studies with and without motivational interviewing to result in similar effect sizes [35]. Two reviews examined the effects of behavioural- and cognitive-type interventions [31,32]: while single type of intervention was not associated with differences in effect sizes, interventions employing a combination of cognitive and behavioural strategies were found to be more effective [31].

Feedback: The impact of feedback was calculated in three reviews with largely differing results [31,35,42]. The two reviews which used CALO-RE taxonomy [35,42] found that providing feedback on performance led to significant differences in effect sizes, but the effect was positive in one review [42] and negative in another [35]. In the third review feedback was not associated with larger effect sizes [31]. One review did not perform calculations related to feedback, but, based on characteristics of included studies, indicated its positive impact on PA outcomes when combined with frequent and focused follow-up [34].

Self-monitoring: All four reviews examined the role of self-monitoring. Two of the reviews reported larger effect sizes of interventions that used self-monitoring than those that did not: in one review this effect was positive [32] and in the other the effect was negative [35].

Modelling: The presence or absence of social or role modelling was not associated with statistically significant differences in effect sizes in two reviews [31,32]. However, the BCTs ‘Model/demonstrate behaviour’ and ‘Provide normative information about others’ behaviour’ led to larger effect sizes in another review [35].

Social support: Social support was not associated with particular effects in two reviews [32,42], but was found to have a negative effect on PA levels in another review [35]. One review did not perform relevant calculations, but highlighted the observation that social support might be an effective component of successful walking interventions [38].

Goal setting: Three reviews [31,35,42] assessed the impact of goal setting and found no statistical difference in effect sizes in interventions which employed these strategies.

Problem solving: Problem solving techniques and barriers management were found to be effective in changing PA behaviour in two reviews [31,35] and no effect was recorded in another [42].

Education: All four reviews assessed the impact of specific educational practices on PA behaviour change and aspects of health education were found to be negatively associated with PA levels in three reviews [32,35,42]. Specifically, using BCTs ‘Provide information on consequences of behaviour to the individual’ and ‘Provide information on where and when to perform the behaviour’ led to larger negative effect sizes in two reviews [35,42].

Prompting: Of three reviews that examined the use of prompts in PA promotion, two did not find significant effects [31,42] and one reported negative effects of prompting on PA levels [35].

Counselling: Stress management techniques and counselling were not associated with larger effect sizes in two reviews that examined these BCTs [31,35].

Other BCTs: In one review [35] two other BCTs were associated with larger negative effect sizes: ‘Action planning’ and ‘Relapse prevention/coping planning’, and one BCT led to positively larger effect sizes: ‘Provide rewards contingent on successful behaviour’. Some other BCTs which were not associated with larger effect sizes are listed in Table 8.

#### Narrative reviews: PA outcomes

All narrative reviews reported some kind of PA outcomes (see Table 9). Most often these were self-reported PA levels, assessed through questionnaires (e.g. CHAMPS, PASE, 7-day PA Recall) and/or activity logs. Three reviews [29,40,44] reported changes in PA levels measured objectively through pedometer or accelerometer and in two reviews it was unclear how the PA levels were measured [46,47]. At least 127 studies, examining interventions of duration between one and 36 months, reported positive outcomes and no change was recorded in at least 26 studies. Three reviews [33,36,40] reported on changes in walking time, speed or distance specifically, based on walking logs (13 studies reported increase of PA and no change was observed in one study).

**Table 9:** Outcomes of interest and timings in the 11 narrative reviews, for which information was available.

Outcomes of interest, effects and timings (narrative reviews)					
outcomes of interest		review	effects and timings	number of studies	summary
physical activity outcomes	PA levels • self-reported (questionnaires, e.g. PASE and the Auckland Heart Exercise Questionnaire, CHAMPS assessment, 7-day PA Recall, activity logs, weekly total time spent in PA, estimated energy expenditure, mean number of occasions of PA, activity intensity) • objective (pedometer, accelerometer, PA duration)	Arbesman 2012 [29]	• improvement at 2mo, 6mo, 9mo and 12mo	6	• improvement: 127 studies (1mo-36mo) • no difference: 26 studies (2wk-24mo)
		Baxter 2016 [30]	• improvement at 6wk, 10wk, 12wk, 13wk, 14wk, 16wk, 25wk, 30wk, 47wk, 73wk, 3mo, 4mo, 6mo, 8mo, 12mo, 24mo, 36-48mo	47	
			• no difference at 6wk, 3mo, 6mo, 8mo, 12mo	8	
		Conn 2003 [33]	• improvement at 3mo, 6mo, 12mo and 24mo	10	
			• no difference at 2wk, 1mo, 1.5mo, 3mo and 24mo	7	
		Geraedts 2013** [36]	• improvement at 12wk and 12mo	4	
			• no difference at 6mo	1	
		King 1998 [39]	• improvement at 12wk, 3mo, 16wk, 20 wk, 6 mo, 9mo, 1yr, 18mo, 24mo, 36mo	16	
		Müller 2014** [41]	• improvement at 1mo, 8wk, 16wk, 24wk, 6mo, 12mo, 24mo	12	
			• no difference at 3mo, 24mo	2	
		Ostrander 2014** [44]	• improvement at 8wk, 12wk, 4mo and 12mo	4	
		Moore 2016* [40]	• improvement at 12mo	1	
		Stevens 2014 [45]	• improvement at 6mo and 12mo	6	
			• no difference at 8mo and 12mo	3	
		Van der Bij 2002 [46]	• improvement at 2.5mo, 3mo, 4mo, 5mo, 6mo, 12mo, 24mo	9	
			• no difference at 6mo, 12mo, 18mo, 24mo	4	
		Van der Deijl 2014 [47]	• improvement at 1mo, 3mo, 6mo, 9mo and 11mo	12	
			• no difference at 3mo, 4mo, 6mo, 12mo	5	
	PA levels • walking time/ speed/ distance (walking logs, self-reported walking times)	Conn 2003 [33]	• improvement at 9wk, 3mo, 6mo and 12mo	5	• improvement: 13 studies (8wk-30mo) • no difference: 1 study (12mo)
			• no difference at 12mo	1	
		Geraedts 2013** [36]	• improvement at 8wk, 12wk and 5mo	3	
		Müller 2014** [41]	• improvement at 8wk and 12wk	2	
		Ostrander 2014** [44]	• improvement at 12wk	1	
		Moore 2016* [40]	• improvement at 12mo and 30mo	2	
psychological outcomes	• self-efficacy	Arbesman 2012 [29]	• small short-term improvements (from meta-analysis and 1 study)	2	• improvement: 11 studies (4mo-24mo) + 1 meta-analysis (short-term, small to medium-sized effect) • not sustained in 2 studies (12mo and 36mo) • no difference: 2 studies (16wk and 6mo) • decrease: 2 studies (12wk)
			• improvement at 4mo, 12mo and 24mo	2	
			• improvement at 12mo and 24mo but not at 36mo; at 6mo but not at 12mo	2	
		King 1998 [39]	• improvement at 8wk and 6mo	1	
		Baxter 2016 [30]	• improvement at 5mo and 10mo	2	
			• no difference at 16wk and 6mo	2	
			• decrease in 1 study	1	
		Moore 2016* [40]	• improvement at 12mo	1	
		Ostrander 2014** [44]	• decrease at 12wk	1	
		Stevens 2014 [45]	• improvement at 12mo	1	
	• quality of life (SF-36 most often, also Vitality Plus Scale)	Arbesman 2012 [29]	• improvement at 6mo and 12mo	2	• improvement: 9 studies (11wk-24mo) • no difference: 3 studies (12mo) • decrease: 1 study (12mo) • small effect sizes for physical function, general health and anxiety; small to moderate effect sizes for social function, energy levels and change in health; moderate effects for aspects of mental health and impact on daily activities
			• no difference in 1 study	1	
		Baxter 2016 [30]	• improvement in 2 studies	2	
		Foster 2013** [34]	• improvement at 12mo and 24mo	2	
			• no difference at 12mo	1	
		King 1998 [39]	• improvement at 6mo	2	
		Moore 2016* [40]	• improvement at 11wk	1	
		Stevens 2014 [45]	• improvement at 12mo	1	
			• no difference at 12mo	1	
			• decrease at 12mo	1	
	• depressive symptoms / mental outlook	Arbesman 2012 [29]	• decreased depressive symptoms at 6mo and 12mo	2	•improvement: 3 studies (6mo-12mo) • no difference: 1 study (10wk)
		Baxter 2016 [30]	• decreased depressive symptoms at 12mo	1	
		Moore 2016* [40]	• no difference at 10wk	1	

* face-to-face interventions only

** remote interventions only

#### Narrative reviews: Psychological outcomes

Seven reviews [29,30,34,39,40,44,45] reported on psychological outcomes pertaining to PA, including: self-efficacy, quality of life, and depressive symptoms.

Self-efficacy was reported in six reviews [29,30,39,40,44,45]. Improvements in self-efficacy were noted in eleven studies measuring effects at 4 months to 2 years [29,30,39,40,45], however this effect was not sustained in two studies beyond 12 months and 3 years [29]. One review reported decrease in self-efficacy at 12 weeks in one study [44].

Quality of life was equally often reported outcome [29,30,34,39,40,45], usually measured with the use of SF-36 questionnaire, consisting of sub-scales primarily relating to health and functional outcomes. Improvements in quality of life, based on the SF-36 scale, were noted in nine studies at times varying between 11 weeks and 12 months [29,30,34,39,40,45]. No difference was observed in three studies [29,34,45] and one study reported a decrease in quality of life at 12 months [45]. One review [39] reported improvements in all SF-36 sub-scales and improvements in the vitality sub-scale were noted by four other reviews [29,30,40,45]. Other sub-scales with improved scores included: physical functioning, social functioning, mental health [29,30,34,40], general health perceptions [29,34,45], bodily pain and energy/fatigue [29,30,34].

Outcomes related to depression and general ‘mental outlook’ were reported by three reviews [29,30,40]. Decreased depressive symptoms were noted in three studies at six and 12 months [29] and in one study no change was observed at 10 weeks [40].

#### Narrative reviews: Participation and adherence

Initial and sustained participation in PA or adherence to interventions promoting PA were reported in five reviews [29,36,39,46,47]. Participation rates were reported in at least 40 studies included in these reviews and varied substantially from 36% to 100%. Some reviews reported the mean participation rate, which ranged from 72% to 79.2%.

#### Narrative reviews: Intervention characteristics

The majority of narrative reviews attempted to establish the characteristics of most effective interventions and those that do not seem to affect outcomes. Four reviews highlighted the importance of tailoring the interventions to participants [29,37,40,44], particularly by environmental mediators, personal readiness and interests and type of activity available locally [37,44]. Environmental suggestions may include tailored information about opportunities in the local environment [37], for example maps of walking or cycling routes, information about upcoming events, neighbourhood gyms and exercise that could be done at home [44]; it seems particularly important that these environmental mediators match individual interests of older adults [44].

Mode of delivery did not appear to be a differentiating factor [37,44], as both face-to-face [40] and remote interventions [41] and group and individual delivery [33] led to generally positive outcomes. However, one review indicated that a supervised home-based format, or a combination of group- and home-based formats could be more effective than a class or group format only [39]–finding not consistent with results of meta-analyses discussed earlier. One review highlighted particular effectiveness of interventions promoting low- to moderate- intensity activity [40], while another concluded that outcomes were unrelated to intensity of frequency of PA [46]. Frequency of intervention (meaning number of intervention contacts) did not seem to affect PA engagement in another review [37].

Behavioural or cognitive-behavioural strategies were reported to have led to more positive outcomes than health education or instruction alone [39]. Interventions using remote feedback were more effective than treatment as usual and equally effective as supervised exercise without feedback [36]. Positive impact of having an activity partner if preferred [44] was also highlighted.

## Strengths and limitations

To our best knowledge, this is the first such extensive and comprehensive review of systematic reviews of PA promotion aimed at older adults and the first to report on both effectiveness and components of the most successful interventions, as well as on impacts of interventions on PA levels and psychological outcomes. Through our inclusive approach to PA promotion activities, which was not limited to only behavioural and cognitive strategies, we were able to highlight a range of factors that seem important to consider when designing programmes to improve PA among older adults.

Despite every effort to gather the best evidence available, this work has inevitable limitations. Firstly, scarcity of high quality evidence syntheses on the theme led to our decision to analyse data from all 19 reviews identified, of which a majority were at high risk of bias. This decision unavoidably impacts on the quality of evidence summarised in the current review, which should be interpreted with caution. Secondly, significant heterogeneity of studies, outcomes and intervention types did not allow us to conduct any statistical analyses beyond reporting of results from meta-analyses performed by authors of included reviews. Throughout the process we relied on evidence as presented by those reviewers; we examined any data tables within the reviews but did not consult individual studies [52]. It is also worth mentioning that most of the included reviews based their calculations or syntheses on self-reported PA outcome measures, which, although considered the most feasible way of collecting data from large populations [53], are prone to a number of biases and human errors [54]. Moreover, different classification systems of BCTs were used in the included reviews, making comparisons particularly difficult. Although the CALO-RE taxonomy [49] provided a useful framework in two of the reviews, we decided not to use any particular nomenclature and a simplified classification was adopted for the purpose of this work to accommodate all BCTs mentioned in the reviews. Lastly, as most of the reviews were narrative and mixed results were typically reported by individual reviews and studies, definite conclusions cannot be drawn about the overall strength of evidence and the effectiveness of the BCTs and other intervention components.

## Discussion

The current review suggests that multi-modal and multi-component interventions have the potential to be effective at increasing physical activity of older adults living in the community. However, heterogeneity of interventions and a high chance of bias in the included reviews made it difficult to assess with certainty the precise magnitude of these effects. We were particularly interested in evidence of long-term effects, and the review demonstrates some positive and sustained effects of interventions on PA levels, self-efficacy and quality of life. Crucially, effects on maintenance beyond twelve months were unclear, due to a lack of high quality longitudinal studies, noted also in other reviews of PA promotion [23,55]. The generally positive effects we recorded were of small to moderate sizes, suggesting a potential impact at the population level, despite challenges in designing, implementing and researching interventions for large ageing populations. Our findings are in line with results reported by recent reviews of interventions to promote PA for adults [55] and those focusing on PA promotion in primary care [18,56] and contribute to a growing evidence that sustained PA participation of older adults may be successfully promoted.

The current review focused primarily on PA-related outcomes including self-reported and objective PA levels, but we were also able to observe some impact of the interventions on outcomes related to psychological wellbeing. There is now a robust empirical evidence that engagement in PA can improve mental wellbeing of older adults [6,7,57,58] and indeed reduce symptoms [59] and prevent recurrence of depression [60]. In our review interventions generally seemed to lead to improved self-efficacy but there was less consistency in their impact on the quality of life and mood-related outcomes. It is worth noting that most studies that failed to observe improvements in the last two areas assessed short-term changes, typically under 12 weeks from the start of the intervention. While no significant changes in quality of life or mood were found in a meta-analysis interested in immediate post-intervention measurement [O], other reviews generally reported improvements at six or twelve months, suggesting that longer intervention and/or follow-up times might be needed to observe positive effects of PA on psychological wellbeing related to mood and quality of life.

### Intervention characteristics

Despite difficulties with comparing heterogeneous interventions, we were able to identify intervention characteristics that seem to contribute to successful PA promotion among older adults and intervention features that do not seem to influence PA uptake or maintenance. Rarely were the intervention components consistently associated with positive or negative findings, most often producing mixed results.

Both multi-modal interventions and interventions employing a single specific delivery mode tended to result in increased PA. In line with another relevant review not meeting the criteria for inclusion in the current synthesis [61], we failed to establish with certainty, due to insufficient evidence, whether face-to-face or remote delivery achieves the most positive results. For example, health coaching achieved better effects when delivered face to face than via telephone, however the importance of face to face delivery might not be transferable to other interventions for PA promotion. Overall, the evidence suggests that interventions of any modality are more effective at increasing PA than no intervention.

Similarly, neither delivery setting nor mode (group or individual) in itself seem to influence outcomes–a finding in line with that of another review of reviews focused on PA promotion for adults [20]. However, the literature reviewed seems to indicate that the relationship between the setting and individual/group delivery mode may indeed influence outcomes. The nature or direction of this effect remains unclear. Some authors [41] argue, for example, that in specific settings (including care homes) non-face-to-face interventions may need to be complemented by in-person contact to enable personalisation according to individual care needs. Future research should specifically investigate the combinations of delivery mode and setting for optimal effectiveness and sustained PA.

In line with other reviews professional background of those who deliver the intervention did not seem to influence outcomes [20]. However, frequency of contacts with the intervention provider may influence effectiveness, but there is no clarity on optimal type and number of contacts. Future research should, therefore, examine not only the frequency and duration of contact, but also other characteristics of those who initiate or implement interventions, including, for example, empathy or self-confidence. Instructor qualities, training and personality have been shown to influence attendance to exercise classes [62] and may be important factors to further examine in community settings and promotion of self-administered PA.

Although type of PA does not seem to influence effectiveness, there is indication that low- to moderate- intensity interventions may be preferred in the age group concerned. Walking-based interventions are particularly common in PA programmes for older adults, due to universality and high acceptability of walking in this population [63]. While we could not test the effectiveness of walking against other types of PA, walking seemed to be particularly efficacious in one review [38], with positive effects stronger than effects reported by other reviews focusing primarily on multi-modal interventions, suggesting that limiting choice by promoting a specific acceptable type of PA may be particularly beneficial for older adults–a notion worth exploring in further research.

Tailoring of interventions to participants’ needs appears to be an important element of successful PA programmes. Positive effects of individualised interventions have been indicated in a number of high quality studies and reviews [55,64,65,66,67]. While our review confirms the already robust evidence that person-centeredness is important for enhanced benefits of PA promotion, we found that direct contact with participants does not seem necessary while tailoring and individualisation seems to be more beneficial when focused on environmental mediators rather than on psychosocial mediators alone.

### Intervention components

As interventions to promote PA seem to generally focus on behavioural and/or cognitive strategies, we were able to establish, to some extent, which BCTs lead to desirable outcomes for older people. Effective interventions typically utilised behavioural, motivational and/or cognitive-type components as opposed to health education or instruction alone [68]. However, a recent review of interventions to reduce sedentary behaviour (SB) in adults [19] found, in contrast to our findings, a beneficial influence of education on SB outcomes, indicating that successful reduction of SB and promotion of PA may require a different approach, or indeed that a technique generally successful in younger populations [69] may not be suitable for older adults. While older people form the most sedentary segment of the population [70], further research in the area is recommended.

It is probable that in order to engage with PA older adults require motivation derived from benefits other than purely cognitive increase of knowledge–a phenomenon widely explored in theories of motivation (e.g. [71,72]). Indeed, an in-depth study using focus groups to establish motivations of older people to engage in PA [73] found that the participants already felt superior to younger peers with regards to knowledge relating to behaviour, habits and health. Similarly, a recent review [74] established that older adults already reported substantial awareness that PA was important to maintain health and improve mood or relieve stress.

The benefits of several other BCT components seem to be uncertain, including prescribing exercise, prompting individuals to practice, providing information on consequences of behaviour, and instructing where and when to practice. Evidence for provision of feedback as a BCT seems contradictory [75]. O’Brien and co-authors [42] consider potential combined effects of self-regulatory techniques, having found that feedback typically concurs with at least one other BCT, leading to positive results which perhaps could not be attributed to feedback alone. We suggest that ongoing follow-up on progress and individualised feedback is a promising strategy for PA promotion, but may need to be carefully adapted to the needs of older adults and potentially combined with other techniques for significant benefits.

Among a number of self-regulatory BCTs examined in this review, generally producing mixed results, barrier identification and problem solving were the techniques most consistently linked to positive outcomes. In contrast, goal setting consistently did not seem to affect the effectiveness of interventions. It may be that goal-setting is too strongly linked to other self-regulatory techniques to be effective in its own right and future studies may set out to examine the relationships and potential combined effects of BCTs which are likely to be applied together in a single intervention.

The mixed results question suitability of self-regulatory strategies for older adults. While other systematic reviews of interventions to promote PA directed at adults generally report positive effects of BCTs [19,20,67,75,76], it appears that techniques successful with younger populations are not necessarily transferable to older adults. Our findings support the conclusions drawn by French and co-authors [35], who additionally ponder if self-regulatory BCTs may be too complex in circumstances of declining functioning. A recent large RCT examined the questionable benefits of self-regulatory techniques [77] and proposed ways of personalising interventions to the ageing population needs by mapping specific BCTs onto the physical capabilities and utilising a more flexible approach. Warner et al. [77] argue that it might be appropriate to decrease the cognitive burden of self-regulatory tasks and emphasise physical activities as opportunities to experience positive affect. It is important to highlight that tailored BCTs are more likely to meet personal needs and respond more accurately to the individual’s position in the behaviour change cycle. Thus, we suggest that self-regulatory BCTs may be beneficial in PA promotion, but need to be more thoroughly examined to utilise their potential for older adults.

### Intervention context

Older adults’ engagement in PA may, therefore, benefit from other motivators, for example social support, which certain types of intervention naturally offer. While high profile studies and reviews report that social contact and support were important facilitators of participation in PA, particularly relevant to ageing populations [66,73,74,77], we were not able to confirm this effect with certainty. Indeed, a strategy involving planning of social support seemed counter-effective in promoting PA. However, there were indications that the social aspect of walking activities might have improved participation and that having an exercise partner, if preferred, could lead to better outcomes. In light of these inconsistencies, a more in-depth exploration of the value of social support for increasing PA in older adults is essential, including its potentially differing role for both initiation and maintenance of PA, as indicated by Van Stralen and co-authors [78].

The literature reviewed also points to the potentially beneficial role of environmental mediators. Although in this review we were not able to assess the indicated positive influence of environmental determinants, other literature provides more support of this notion [19,79], including systematic reviews highlighting the importance of the environment for the uptake and maintenance of PA in ageing population [74,78]. Alongside social support and enabling environmental factors, literature tends to mention enjoyment as a motivator for older adults to undertake and sustain PA [73,77]. One of the reviews we examined highlights the value of enjoyable and sociable activities [35] and other reviews, not meeting the inclusion criteria for our synthesis, found a positive association between enjoyment of being physically active and both initiation and maintenance of PA [78], concluding that group-based activities were particularly valued for instilling a sense of belonging, opportunities for friendship and, ultimately, enjoyment [74]. Moreover, a recent review which explored acceptability of the concept of being physically active [80] found that PA was often perceived by older adults as a by-product of other more purposeful activities. Further research may, therefore, explore in more detail the role of enjoyment and purpose for PA uptake and maintenance; both concepts seem crucial in designing acceptable and meaningful interventions to promote PA among ageing population.

In summary, we believe that there is an urgent need to look beyond psychological and cognitive theories of behaviour change towards whole system-oriented approaches [81,82], including environmental and social aspects of PA promotion, in order to understand what activities older people like to engage in and how professionals can support this engagement [83]. Too often are public health interventions developed using a top-down approach and effectively isolating the end-user. In our review, theory-driven interventions were dominating and none of the included reviews looked at interventions co-created with their recipients, despite the growing evidence that including older adults as major stakeholders in the design process could lead to more acceptable and appropriate interventions and, in effect, to sustained behaviour change [84,85]. While the benefits of co-creation in healthcare service development are now increasingly more often recognised [82,86], future research may explore the role of co-creation in designing PA promotion strategies for older adults. Equally important is ensuring that the interventions address the needs of those whose inactivity may be associated with health inequalities due to the social or economic background, environmental exposure and disability [87,88]—factors which combined with older age pose particular challenges for engagement in PA.

## Research recommendations

The growing recognition of the importance of PA promotion for older adults is worth emphasising, as are the latest attempts to synthesise the emerging evidence. Astonishingly, a large majority of the papers included in this review (15 out of 19) were published in the last five years (after 2012). Two reviews of reviews on PA among older adults [22,23] have been published very recently, parts of which focus on PA promotion. Olanrewaju et al. [23] included 17 reviews, reporting on short term effects of both PA promotion and exercise and Bauman et al. [22] presented a brief synthesis of outcomes of 6 reviews. Both reviews were narrative and had a wider focus on PA among older adults, including the effects of exercise, with PA promotion explored as part of a larger investigation, and thus not as systematically examined as in the current review. An added value of our review is its focus on long term effects, assessment of psychological outcomes, inclusion of several new, key and high quality reviews (e.g. [30,34,42,43]), and the most comprehensive analysis of reviews focusing purely on interventions to promote PA and not exercise alone. Despite our and our colleagues’ efforts in synthesising the growing evidence, unanswered questions remain.

We recommend that future research studies explore the potential of PA promotion interventions to effect sustained improvements. Large scale longitudinal projects with follow up beyond two years are much needed to identify the interventions capable of achieving long-term results and establish ways to maintain engagement with PA. Further research into intervention components most beneficial for older adults is also required. Future research studies might want to focus on ways to adapt the BCTs currently successful with adults to better meet the needs of ageing populations. The influence of motivators, such as social contact, opportunities in local environment, and enjoyment, should be further explored.

Implementation processes will have to take account of barriers to implementation at the level of individual older people, professionals and their practice and organisational systems and processes. Future research needs to emphasise systemic and contextual factors of interventions. Overcoming the narrow focus on BCTs is crucial for identifying the most successful features of interventions in most enabling contexts. The recommended shift towards researching even more complex intra- and interpersonal as well as environmental factors influencing uptake and maintenance of PA may require more interdisciplinary approaches and development of more suitable and comprehensive process and outcome measures.

## Conclusions

Despite challenges around summarising evidence from heterogeneous studies and reviews that generally presented high risk of bias, the current review confirms the effectiveness of multi-modal and multi-component interventions to promote physical activity for older adults. Although the effect sizes were small to moderate, interventions generally led to increased PA and to improvements in outcomes related to wellbeing. Mode of delivery, setting and profession of the intervention provider are not necessarily associated with effectiveness, but client-centred, personalised interventions which start with professional and tailored guidance and then provide ongoing support lead to improved participation in PA. Our review highlights the mixed results around the role of self-regulatory BCTs in PA promotion among older adults and advises further research to establish whether and how the potentially beneficial BCTs may be adapted to meet the needs of ageing populations. There are indications that purely cognitive strategies and BCTs requiring active planning might be less suitable for older adults than motivators more meaningful in their current lives, including social support, environmental factors and enjoyment coming from being physically active.

## Supporting information

S1 Checklist PRISMA checklist. (DOCX)

S1 Table Overview of the 17 included reviews. (XLSX)

S2 Table Intervention characteristics in the 17 included reviews. (XLSX)

S3 Table Search string. (DOCX)

## Abbreviations

BCT: behaviour change technique
PA: physical activity
RCT: randomised controlled trial
