# Supplementary material for: Promotion of physical activity interventions for community dwelling older adults: A systematic review of reviews
Source: PLoS One. 2017 Jul 10;12(7):e0180902. doi: 10.1371/journal.pone.0180902 (PMC5507305; doi:10.1371/journal.pone.0180902)
Supplement: S3 Table — (DOCX) [file pone.0180902.s004.docx]

Search string

| S1 | (MH "Physical Activity") or (MH "Exercise+") or (MH "Physical Mobility") |
| --- | --- |
| S2 | (MH "Systematic Review") |
| S3 | (MH "Literature Review") |
| S4 | TX ((Promot* or uptake or encourage* or increase* or start) n2 (physical activity)) or ((Promot* or uptake or encourage* or increase* or start) n2 (exercise)) or ((Promot* or uptake or encourage* or increase* or start) n2 (aerobics or circuits or smimming or aqua*)) or ((Promot* or uptake or encourage* or increase* or start) n2 (jogging or running or cycling)) or ((Promot* or uptake or encourage* or increase* or start) n2 ((keep fit) or (fitness class*) or yoga)) or ((Promot* or uptake or encourage* or increase* or start) n2 walking) or ((Promot* or uptake or encourage* or increase* or start) n2 sport*) or ((Promot* or uptake or encourage* or increase* or start) n2 gardening) or ((Promot* or uptake or encourage* or increase* or start) n2 housework) |
| S5 | (MM "Aged") |
| S6 | TI elderly or old age or older |
| S7 | AB old or older age or over 50 or over fift* or over 55 or over fifty five* or over sixt* or over 60 or over sixty five* or over 65 or over sevent* or over 70 or Over eight* or over 80 or over ninet* or over 90 or over ninety five or over 95 |
| S8 | (MH "Review Literature as Topic") OR (MH "Review") |
| S9 | TI review |
| S10 | DE "Literature Review" |
| S11 | S2 OR S3 OR S8 OR S9 OR S10 |
| S12 | S1 OR S4 |
| S13 | S5 OR S6 OR S7 |
| S14 | S11 AND S12 AND S13 |
| S15 | DEDUPLICATE |
